# Structural Analysis of Hand Drawn Bumblebee *Bombus terrestris* Silk

**DOI:** 10.3390/ijms17071170

**Published:** 2016-07-20

**Authors:** Andrea L. Woodhead, Tara D. Sutherland, Jeffrey S. Church

**Affiliations:** 1 CSIRO Manufacturing, Pigdons Road, Waurn Ponds, VIC 3216, Australia; andrea.woodhead@csiro.au; 2 CSIRO Food and Nutrition, Clunies Ross Street, Black Mountain, ACT 2601, Australia; tara.sutherland@csiro.au

**Keywords:** silk, protein conformation, protein orientation, Raman spectroscopy, *Bombus terrestris*, coiled coil, fibre

## Abstract

*Bombus terrestris*, commonly known as the buff-tailed bumblebee, is native to Europe, parts of Africa and Asia. It is commercially bred for use as a pollinator of greenhouse crops. Larvae pupate within a silken cocoon that they construct from proteins produced in modified salivary glands. The amino acid composition and protein structure of hand drawn *B. terrestris*, silk fibres was investigated through the use of micro-Raman spectroscopy. Spectra were obtained from single fibres drawn from the larvae salivary gland at a rate of 0.14 cm/s. Raman spectroscopy enabled the identification of poly(alanine), poly(alanine-glycine), phenylalanine, tryptophan, and methionine, which is consistent with the results of amino acid analysis. The dominant protein conformation was found to be coiled coil (73%) while the β-sheet content of 10% is, as expected, lower than those reported for hornets and ants. Polarized Raman spectra revealed that the coiled coils were highly aligned along the fibre axis while the β-sheet and random coil components had their peptide carbonyl groups roughly perpendicular to the fibre axis. The protein orientation distribution is compared to those of other natural and recombinant silks. A structural model for the *B. terrestris* silk fibre is proposed based on these results.

## 1. Introduction

Over recent years, there has been a growing interest in natural and synthetic insect silks for their physical properties and biocompatibility. The aculeates (Hymenoptera, stinging insects) are an abundant group of insects that includes the social species of ants, hornets, and bees. The larvae of many of these insects produce coiled coil silk proteins in modified salivary glands that they fabricate into materials for a range of applications extending from individual cocoons to extensive communal domiciles [1]. Often the silk is used in conjunction with other materials. For example, bee hives are a composite of wax and silk and hornet nests combine silk and plant fibres.

The aculeate silk proteins are characterized by having primarily a coiled coil molecular structure, a structure that is indicated by bioinformatics analysis of the proteins primary amino acid sequence and confirmed by experimental analysis [1,2,3,4,5,6,7]. While the proteins are largely coiled coil in conformation, significant level of β-sheet structure has been detected by X-ray diffraction, infrared, and/or solid state NMR analysis in the silks of native bees, ant and hornet silk, reconstituted hornet silk, and artificial honeybee silk. The greatest proportion of β-sheet structure is found in hornets with moderate levels in the ants and the least amounts in the bee lineages [1,2,3,4,5,6,7].

*Bombus terrestris,* also known as the buff-tailed bumblebee or large earth bumblebee, is one of the most abundant bumblebee species in Europe. Since 1987 *B. terrestris* has been bred commercially for use as a pollinator for European greenhouse crops [8,9]. It has been commercially reared in New Zealand since the early 1990s [10,11] and is now used in many countries and regions including North Africa, Japan, Korea, and Russia [12]. In some countries, including mainland Australia, *B. terrestris* is classified as a feral or invasive alien species, presenting a significant risk to native fauna and flora. The queen is 2–2.7 cm long while workers range from 1.5 to 2 cm in length, both exhibiting a buff-white tail tip. Nests are usually established underground, often in the abandoned burrows of small rodents. Eggs, laid by the queen, hatch yielding larvae which at the end of the final instar spin silk cocoons and pupate. The silk proteins are produced by labial (modified salivary) glands.

Vibrational spectroscopy, and in particular Raman spectroscopy, has been shown to be a valuable technique for studying insect silk and silk worm fibres [13,14,15,16,17,18]. The technique can provide a wealth of information on specific amino acids as well as protein secondary structure and its alignment with respect to the fibre axis. In this paper, we use micro-Raman spectroscopy to investigate the amino acid composition, protein conformation, and orientation of hand drawn native bumblebee, *B. terrestris*, silk fibres. The protein orientation distribution is compared to those of other silks produced by silkworm larvae (*Bombyx mori* and *Sami cynthia ricini*), a golden orb weaver spider (*Nephilaedulis*) and raspy crickets (*Apotrechus illawarra*), as well as that produced by wet spun recombinant honeybee AmelF3 protein. A model for the protein structure of a typical *B. terrestris* silk fibre is proposed.

## 2. Results and Discussion

### 2.1. Microscopic Observations

**Figure 1:**
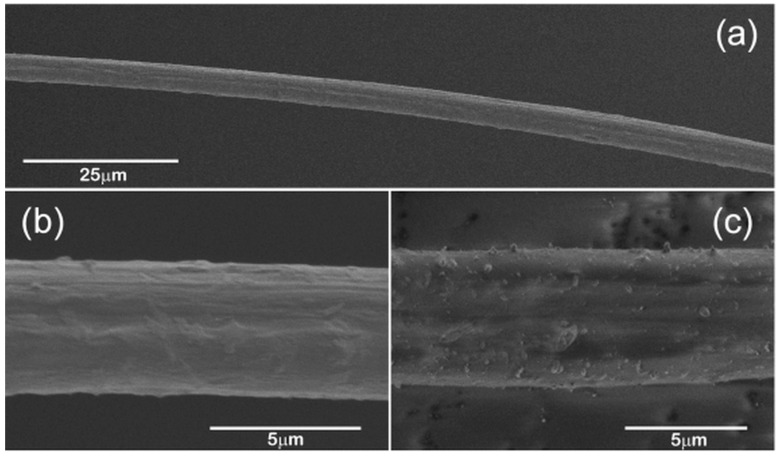
Secondary electron images obtained from (**a**,**b**) section of a *B. terrestris* silk fibre hand drawn at 0.14 cm/s and (**c**) a fibre hand drawn at 0.10 cm/s.

Secondary electron images obtained from hand drawn *B. terrestris* silk fibres are shown as Figure 1.

Low magnification examination of a fibre drawn at 0.14 cm/s (Figure 1a) reveals a monofilament with a relatively uniform diameter, 4.7 ± 0.2 μm, over the 130 μm long section imaged. A slight birefringence was observed for this fibre so it was chosen for Raman analysis. At higher magnification (Figure 1b), a slight surface patterning can be observed. Energy dispersive X-ray (EDX) analysis of the fibre (Figure S1) revealed that the fibre was comprised largely of carbon and oxygen as expected for a protein considering the difficulty of detecting nitrogen using this technique. Weak peaks for sodium, phosphorus, sulphur, chlorine, and potassium were also detected. Similar elements were found in the silk of hornets [19].

An image of the first fibre drawn from the bumblebee is shown as Figure 1c. The diameter of this fibre was determined to be 5.6 ± 0.1 μm. The thicker diameter may be a reflection of the slower draw rate (0.10 cm/s) used when collecting this fibre. The image obtained from this fibre is interesting in that a fine particulate material appears to be embedded into the fibre surface suggesting that the fibre was still in a viscous state when the contact was made. EDX analysis showed that this material (Figure S2) has a composition rich in silicon, aluminium, magnesium, calcium, and iron. Silicon, aluminium, and magnesium have been detected in the silk of weaver ants [20]. However, considering the particulate nature of these deposits, their presence may be associated with a silicate clay, probably on the bumblebee when the first fibres were collected. The particles have become embedded in the still semi-fluid silk protein.

### 2.2. Raman Spectra

**Figure 2:**
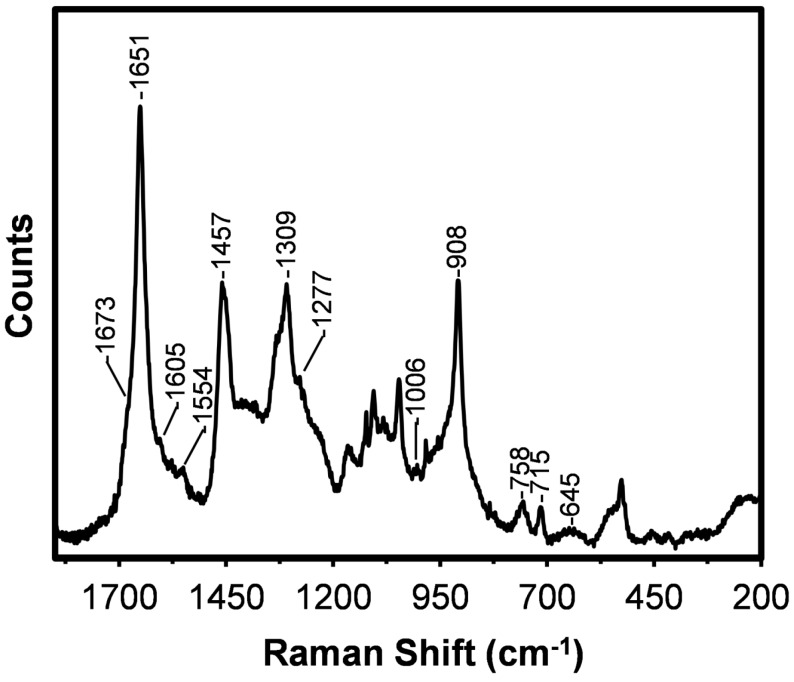
Raman spectrum obtained from the hand drawn (0.14 cm/s) *B. terrestris* silk fibre.

The low frequency Raman spectrum obtained from the *B. terrestris* silk drawn at 0.14 cm/s is shown as Figure 2. The frequencies, relative intensities, and tentative assignments of the bands based on the literature [6,13,21,22,23,24,25] are presented in Table 1.

The spectrum obtained from the *B. terrestris* silk is dominated by a sharp amide I band with a peak maximum at 1651 cm^−1^ which can be assigned to coiled coil protein conformation [6,22]. The coiled coil confirmation is in agreement with bioinformatics analysis of the protein sequence [1]. A weak shoulder on the amide I band near 1673 cm^−1^ is assigned to β-sheet protein [6,13,22]. The dominance of the coiled coil structure is supported by the moderately intense amide III and C–C skeletal (protein backbone) stretching modes observed at 1309 and 908 cm^−1^, respectively [22]. A very weak shoulder on the amide III mode at 1277 cm^−1^ can be assigned to random coil protein [21,23] while a very weak band at 1084 cm^−1^ can be attributed to C–C skeletal stretching of random coil and β-sheet proteins [23]. As well as protein conformational information, specific Raman bands can provide information about the presence of different amino acids, particularly those with aromatic and sulphur-containing side chains. A detailed analysis of these bands, as observed in the *B. terrestris* silk spectrum, is now presented.

**Figure 3:**
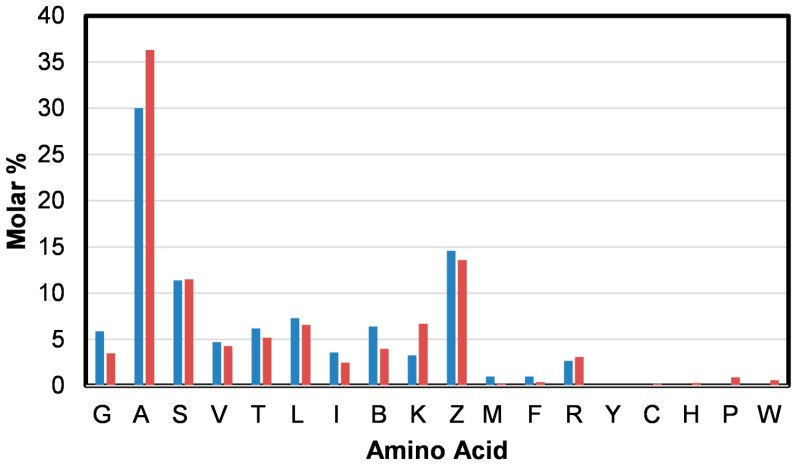
The amino acid composition (molar percentage) determined for *B. terrestris* silk (blue) and determined from amino acid sequences of the four fibrous *B. terrestris* silk proteins; BterF1: ABW21694, BterF2: ABW21695, BterF3: ABW21696, and BterF4: ABW21697 (red) [26].

From the amino acid analysis of the *B. terrestris* silk [26] presented in Figure 3 (blue) it is apparent that alanine (A), glutamic acid and/or glutamine (Z) and serine (S) are dominant. A number of Raman active amino acids, including phenylalanine (F) and methionine (M), are also present in the silk.

Very weak features at 1605, 1576, and 1006 cm^−1^ can be assigned to aromatic ring stretching modes of phenylalanine (F) (Table 1) [22,25]. A weak band observed at 1554 cm^−1^ suggests the presence of tryptophan (W) [22,25] in the silk. This amino acid was not detected (Figure 3 (blue)), possibly because it was destroyed by the protein digestion process utilized. Its presence in the silk is however confirmed from the amino acid sequence analysis shown as Figure 3 (red). Very small amounts of cysteine (C), histidine (H), and proline (P) were also detected by this method. Tryptophan also exhibits bands that overlap with those of phenylalanine observed at 1576 and 1006 cm^−1^ [22,25]. In accordance with the amino acid analysis, the Raman spectrum obtained from the *B. terrestris* silk does not exhibit features at 854 and 829 cm^−1^ which are attributable to the Fermi doublet of tyrosine (Y) [22,25,27].

As shown in Figure 3, *B. terrestris* silk’s amino acid composition is dominated by alanine (A). Over 18% of the residues in the primary protein sequence of the silk proteins are poly(A), including poly(AA) [1]. There is also a significant presence of glycine (G). The moderately strong band assigned to CH_2_ and CH_3_ bending modes observed at 1457 cm^−1^ have been associated with the presence of poly(A) and poly(AG) segments [16]. The potential energy distribution of the weak band observed at 1107 cm^−1^ can include components attributable to the Cα–C_β_ stretch and C_β_H_3_ rocking modes of poly(A) [23,24]. Weak bands observed at 758 and 527 cm^−1^ have been assigned to CH_3_ rocking vibrations and skeletal bending of poly(A) segments [23,24]. These bands further indicate that poly(A) segments are in a coiled coil conformation.

A spectral feature near 700 cm^−1^ is indicative of the presence of methionine (M) and provides conformational information. An additional methionine band is often observed near 655 cm^−1^ [28]. In the spectrum obtained from the *B. terrestris* silk, a weak but sharp band is observed at 715 cm^−1^ which is assigned to a C–S stretching mode of methionine. This frequency is indicative of the C–C–S–C bonds being in the *trans-*conformation. The detection of methionine is consistent with the amino acid analysis shown in Figure 3. A very weak broad methionine band is also observed at 645 cm^−1^. A number of non-amino acid specific Raman bands are observed at lower wavenumber. These bands are assigned to various skeletal vibrations [23].

### 2.3. Protein Conformation

**Figure 4:**
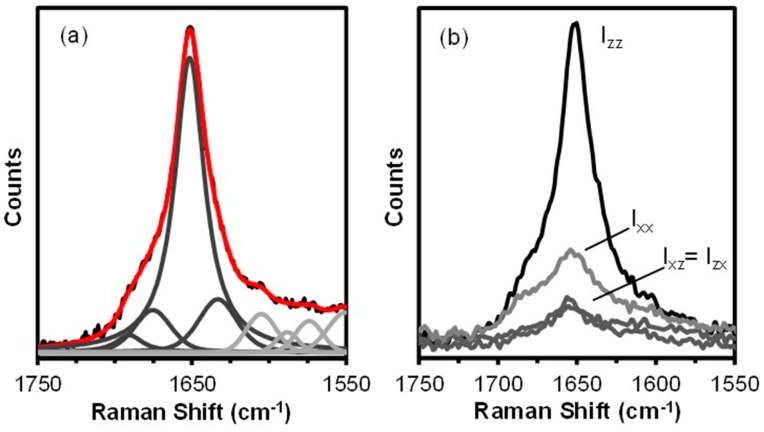
The Raman amide I band of the hand drawn *B. terrestris* fibre. Spectral deconvolution (**a**); the underlying black trace represents the raw data, the red trace is the sum of the component peaks (dark grey) and components that are not associated with protein conformation and present for fitting purposes only (light grey). Polarized Raman spectra (**b**).

The amide I and III regions of the silk spectra provide information about the protein conformations present in the hand drawn *B. terrestris* silk fibre. This information can be enhanced by the deconvolution of the amide band envelopes. The spectral deconvolution of the amide I band was carried out based on component frequencies identified by second derivative spectroscopy and the results are shown as Figure 4a. The band parameters and the conformational composition of the hand drawn *B. terrestris* silk fibre are presented in Table 2.

The spectral deconvolution confirms the dominance of coiled coil structure with lesser components of random coil, β-sheet, and β-turn protein. As expected from the analytical results obtained from bees and hornets [7,26], the 10% β-sheet content determined for the *B. terrestris* fibre is significantly less than the 24% content determined for hornet silk by infrared analysis [27].

As the secondary structural analysis was carried out on the survey spectra, the intrinsic polarization of the laser can lead to errors in the results. Orientation insensitive spectra can be obtained by using a circular polarized laser beam during data collection. Fisk et al. [28] have however proposed a method to obtain an insensitive spectrum from conventional Raman polarized spectra. This method has been adapted for use with micro-Raman polarized spectra by Lefèvre et al. [29]. When this correction is applied, it was found that the β-sheet content presented in Table 2 is slightly underestimated. It is unlikely that the magnitude of this correction is significant.

### 2.4. Protein Orientation

Analysis of Raman amide I and III band polarization measurements provides information about the orientation of the protein chains with respect to the fibre axis. From such measurements observed for the cocoon silks of *B. mori* and *S. c. ricini* and the dragline silks of *N. edulis*, it was found that the dominant β-sheets (amide I maxima at 1665, 1669 and 1668 cm^−1^, respectively) within the fibres are highly orientated parallel to the fibre axis [16]. In contrast, regenerated films cast from solubilized *B. mori* silk and silk protein coagulated in methanol were found to be isotropic [16]. More recently, it has been shown that the protein conformation of fibres produced by two raspy cricket (Orthoptera: Gryllacrididae) species are also dominated by a β-sheet backbone orientated parallel to the fibre axis. On the other hand, polarized Raman spectra obtained from the films observed at fibre crossings suggested that this material was isotropic [13].

The protein conformation and orientation of wet spun recombinant AmelF3 honeybee silk protein was investigated by Poole et al. [30]. Deconvolution of the Raman spectra obtained after injection into the coagulation bath revealed that the structure of the silk fibres was of the order of 36% β-sheet and 31% coiled coil. At this point in production, polarized Raman spectra indicated that there was little if any orientation of the protein chains. The subsequent rehydration and drawing of the monofilament over rollers had little effect on the proportions of the different secondary structures present. There, however, was a significant change in protein chain orientation. One would expect the longer axis of a molecular unit to align with the draw direction. The coiled coils became modestly aligned parallel to the fibre axis. In contrast, the β-sheets appear to take up significant alignment perpendicular to the fibre axis, similar to the ribbons that make up lacewing egg stalk silk [31]. Bauer et al. have shown using polarized Raman spectroscopy that the extension of the egg stalk silk results in the direct conversion of the cross-βsegments into parallel-βsegments [32].

The polarized Raman spectra obtained from the hand drawn *B. terrestris* fibre are shown as Figure 4b. For an isotropic material the parallel (I*_xx_* and I*_zz_*) and crossed-polarized (I*_xz_* and I*_zx_*) spectra are expected to overlapped within experimental error. In the case of the hand drawn *B. terrestris* fibre, the parallel (I*_xx_* and I*_zz_*) spectra are markedly different (Figure 4b) with the I*_zz_* amide I band strongest. This indicates that the coiled coil segments and their associated peptide C=O groups are aligned parallel to the fibre axis.

The level of protein conformational orientation can be quantitatively compared to that of other silk fibres. The theory of orientation measurements by Raman micro-spectroscopy for a Raman tensor with uniaxial symmetry such as that observed in fibres and films has been developed by Bower [33] and Jen et al. [34] and refined for micro-Raman spectroscopy by Turell [35,36]. This approach has recently been applied successfully to protein fibres and films. Based on this theory, the orientation distribution function *N*(θ) of the peptide carbonyl as a function of the angle made with the fibre axis can be represented by an expansion in even term Legendre polynomials,

(1) $$N\left(\theta \right)=\overset{\text{even}}{\underset{\ell }{\sum }}\left(\ell +\frac{1}{2}\right)\cdot \langle P_{\ell }\rangle \cdot P_{\ell }\left(\cos\theta \right)$$

where the first three terms are given by

(2) $$P_{o}\left(\cos\theta \right)=1$$

(3) $$P_{2}\left(\cos\theta \right)=\frac{1}{2}\left(3\cos^{2}\theta -1\right)$$

(4) $$P_{4}\left(\cos\theta \right)=\frac{1}{8}\left(35\cos^{4}\theta -30\cos^{2}\theta +3\right)$$

Only the ‹*P*_2_› and ‹*P*_4_› coefficients, often called order parameters, can be determined using polarized Raman spectroscopy. The method for determining these values from *R*_1_ = *I_zx_*/*I_zz_*, *R*_2_ = *I_xz_*/*I_xx_* and *R*_iso_ = *R*_1_ = *R*_2_ for an isotropic film has recently been presented in detail elsewhere [16,37].

As the orientation distribution function given by Equation (1) is an infinite series, and only the first two non-trivial order parameters can be determined, some information is missing. In many cases, this missing information does not prevent an idea of the true equilibrium distribution from being obtained using this approach. The statistically most probable distribution, *N*_mp_(θ) can be determined through the application of information or Shannon’s entropy theory [38,39]. Through maximizing the information entropy of the orientation distribution as given by

(5) $$S\left[N_{\text{mp}}\left(\theta \right)\right]=-\int_{0}^{\pi }N\left(\theta \right)\ln\left(N\right)\sin\theta d\theta$$

and introducing the Lagrange multipliers, *λ*_2_ and *λ*_4_ one obtains

(6) $$N_{\text{mp}}\left(\theta \right)=\frac{\text{exp}(\lambda_{2}P_{2}(\cos\theta )+\lambda_{4}P_{4}(\cos\theta ))}{\int_{0}^{\pi }\text{exp}(\lambda_{2}P_{2}(\cos\theta )+\lambda_{4}P_{4}(\cos\theta ))\sin\theta d\theta }$$

Once ‹*P*_2_› and ‹*P*_4_› are determined experimentally, the values of *λ*_2_ and *λ*_4_ can be determined numerically using the constraints given explicitly by

(7) $$\langle P_{\ell }\rangle =\overset{\pi }{\underset{0}{\int }}P_{\ell }\cos\left(\theta \right)N\left(\theta \right)\sin\theta d\theta$$

where *ℓ* = 2 and 4.

As a film of the *B. terrestris* silk protein was unavailable, the R_iso_ value determined from the isotropic *A. illawarra* films, 0.20 ± 0.01, was utilized [13]. This value is within experimental error of the values determined for regenerated isotropic *B. mori* and *S. c. ricini* films [16]. The similarity is not surprising as the Raman tensor for the amide I vibration has been found to be independent of backbone conformation [16,40]. The values determined for *R*_1_ and *R*_2_ were 0.13 ± 0.01 and 0.16 ± 0.02, respectively. From these values, a ‹*P*_2_› value of 0.43 ± 0.05 and a ‹*P*_4_› value of 0.16 ± 0.05 were calculated.

**Figure 5:**
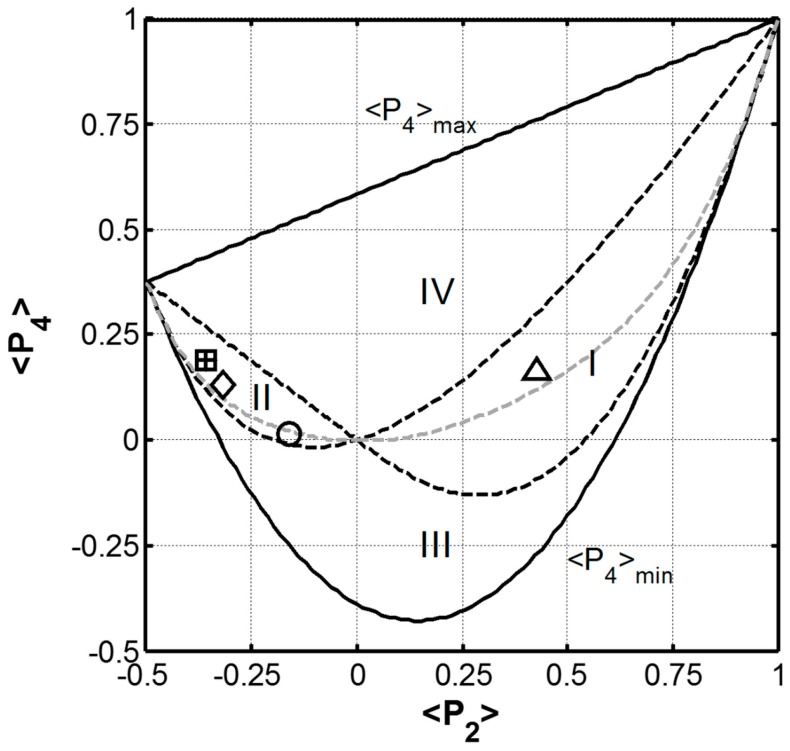
The graph of the limiting values of ‹*P*_4_› as a function of ‹*P*_2_›. The (‹*P*_2_›, ‹*P*_4_›) couple determined for *B. terrestris* (∆), *A. Illawarra* (○) fibres are presented along with those of *B. mori* (+) and *S. c. ricini* (□) cocoon silks and *N. edulis* (◊) dragline silk [13,15]. Note that the values determined for *B. mori* cocoon and *S. c. ricini* cocoon overlap. The dashed grey line represents the values of ‹*P*_4_› when *λ*_4_ = 0.

The combined values of ‹*P*_2_› and ‹*P*_4_›, provide information as to the shape of the orientation distribution function. For a given ‹*P*_2_› value, the allowed ‹*P*_4_› values are limited by Schwartz’s inequality ‹cos^2^*θ*›^2^ ≤ ‹cos^4^*θ*› ≤ ‹cos^2^*θ*›. The (‹*P*_2_›, ‹*P*_4_›) plane for negative and positive ‹*P*_2_› values is shown as Figure 5. The shape of the orientation distribution function for different values of ‹*P*_4_› has been discussed in detail elsewhere [39,41]. Briefly, if ‹*P*_4_› = ‹*P*_4min_›, the orientation distribution function is unimodal and is given by the delta function centered at *θ* = arccos (⅔‹*P*_2_› + ⅓)^½^. When ‹*P*_4_› = ‹*P*_4max_›, the orientation distribution function is bimodal with delta function maxima at 0 and 90°. For all other values of ‹*P*_4_› the distribution function is obtained by analysing the derivative of *N*_mp_(*θ*) which is given as Equation (6).

In Figure 5, the (‹*P*_2_›, ‹*P*_4_›) plane (defined by the solid black lines) can be further divided by the dashed black lines, into four regions identified as I through IV in Figure 5, each with distinct orientation distributions [42]. The (‹*P*_2_›, ‹*P*_4_›) couple determined for the *B. terrestris* silk is plotted in Figure 5 along with those reported for *A. illawarra silk* [13], *B. mori* cocoon, *N. edulis* dragline and *S. c. ricini* cocoon silks [16]. The (‹*P*_2_›, ‹*P*_4_›) couple determined for the hand drawn *B. terrestris* is found in zone I while the other silk fibres all fall into zone II. Zones I and II define the combinations of ‹*P*_2_› and ‹*P*_4_› for which the orientation distribution *N*_mp_(*θ*) shows a monotonic increase with *θ*. The positive ‹*P*_4_› value determined for the hand drawn *B. terrestris* silk indicates that a large proportion of the peptide C=O groups are aligned parallel to the fibre axis as expected for a coiled coil structure running parallel to the fibre axis. In contrast, the negative ‹*P*_2_› values determined for the cricket, cocoon, and dragline silks indicate that a large proportion of their peptide C=O groups are aligned perpendicular to the fibre axis as expected for a β-sheet structure with its backbone parallel to the fibre axis.

**Figure 6:**
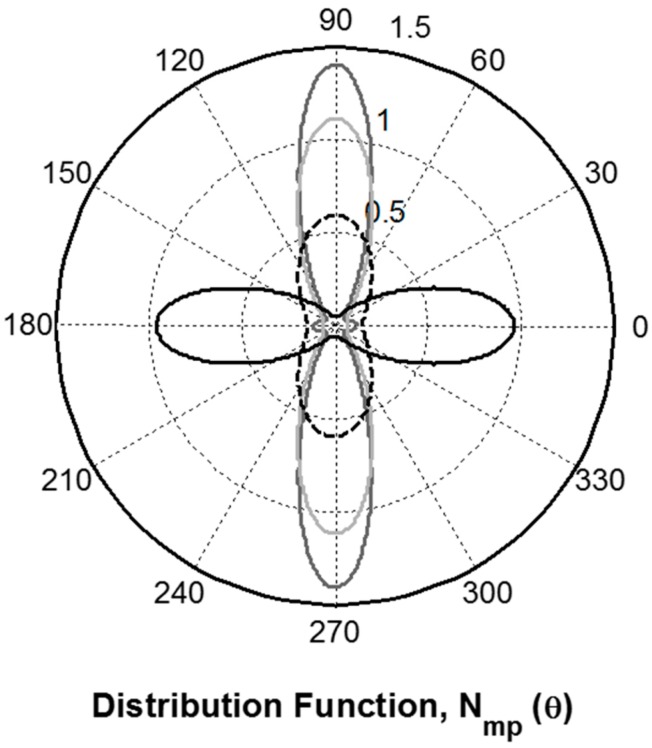
The most probable orientation distribution determined for the hand drawn *B. terrestris* silk fibre (black trace). *A. illawarra* fibres (dashed black trace) [13] presented along with those of *B. mori* and *S. c. ricini* cocoon (dark grey trace) and *N. edulis* dragline (light grey trace) silks [16]. The 0° of the polar plot coincides with the fibre direction.

A third locus of points arises out of the Shannon’s entropy treatment of the orientation distribution function (Equations (5) and (6)) when *λ*_4_ is set equal to zero [41]. This line is also shown as the dashed grey line in Figure 5 and corresponds to a function that is unimodal and Gaussian. The (‹*P*_2_›, ‹*P*_4_›) couples for all of the fibres fall close to this *λ*_4_ = 0 curve indicating that their peptide carbonyl group orientation distributions are all Gaussian. The most probable orientation distribution based on the *λ*_2_ and *λ*_4_ vales determined for the hand drawn *B. terrestris* silk fibre is presented as Figure 6 along with those previously reported for *A. illawarra* silk [13], *B. mori*, and *S. c. ricini* cocoon silk, and *N. edulis* dragline silk [16]. From this representation, it is clear that the carbonyl groups of the hand drawn *B. terrestris* fibre are orientated parallel to the draw axis (0°–180° line) as expected for a coiled coil protein structure.

The most probable probability distributions reveal the likely amounts of peptide C=O groups that are orientated in a given direction relative to the fibre axis. All of the distributions shown in Figure 6 are largely unimodal. Detailed analysis, however, reveals some differences including; the cocoon silks exhibit a minor protein component that is parallel to the fibre axis (small blips on the 0°–180°) and the *A. illawarra* fibres which have a significant amount of randomly orientated protein (non-zero value at all angles). The peptide carbonyl orientation distribution observed from hand drawn *B. terrestris* silk fibre has a similar shape but is at right angles to that observed for the *N. edulis* dragline silk. This observation suggests that the angular probability distribution about the average C=O bond axis for these two silks are very similar.

**Figure 7:**
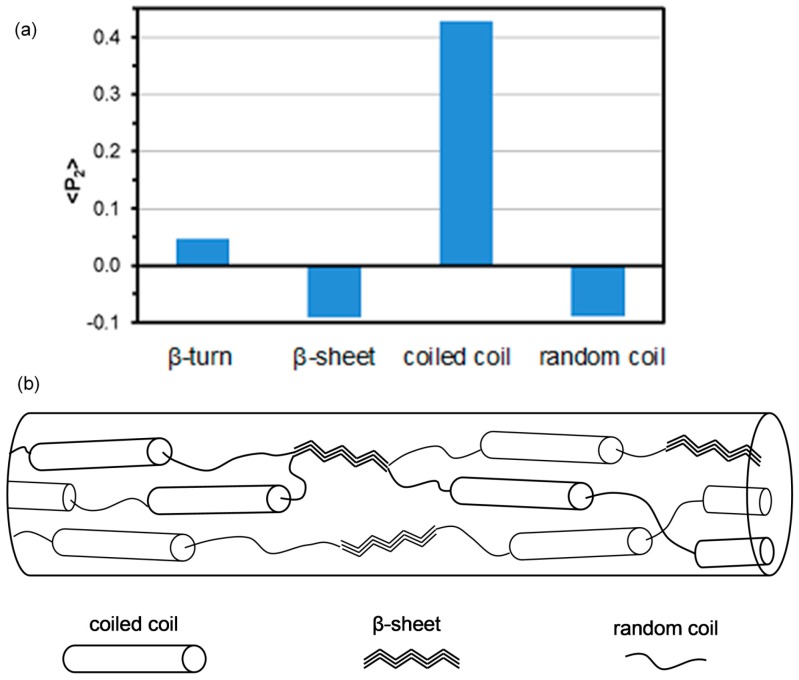
Graph of ‹*P*_2_› values obtained from the deconvolution of the polarized hand drawn *B. terrestris* silk spectra (**a**); Proposed structure of the *B. terrestris* silk fibres based on the Raman results (**b**).

If the polarization spectra is deconvoluted, information on the orientation of the individual protein conformations can be obtained. This approach also removes distortion effects due to component band overlap. Values for ‹*P*_2_› range from +1 for perfect alignment parallel to 0.5 for perpendicular to the draw axis. The ‹*P*_2_› values obtained for the different protein conformations as shown in Figure 7a suggests that all protein conformations exhibit some degree of orientation. This is consistent with dragline and cocoon silks [15]. For the hand drawn *B. terrestris* silk, the coiled coil protein segments have their carbonyl groups rather highly orientated parallel to the draw axis while, for the β-sheet and random coil components of the proteins, the carbonyls are very loosely orientated perpendicular to this axis. Size estimates of the coiled coil predicted by bioinformatics analysis of the primary amino acid sequence suggest a coiled coil unit of 31 nm (210 amino acids) by 2.5 nm. This rod like structure would be expected to orient such that the longest dimension would be in the direction of protein solution flow and subsequent fibre draw. Based upon this analysis, one can propose a structure for the hand drawn *B. terrestris* silk as depicted in Figure 7b.

The structure of *B. terrestris* silk fibres, shown as Figure 7b, is significantly different from that observed for the wet spun recombinant honeybee silk protein [30]. As discussed above, the backbone of the β-structures in this latter silk are aligned perpendicular to the fibre axis. It should be pointed out that the drawing process in this production process involves quickly rehydrating the dried monofilament in a methanol/water bath followed by drafting on a series of draw rollers. Further to this the native silk is drawn from a mixture of at least four proteins while the recombinant silk is produced from only one. This mechanism does not mimic the natural draw process and thus could potentially result in a significantly different final structure. It has been shown for lacewing egg stalk silk that upon extension, the naturally occurring cross-β structure can be transformed into a β-structure that is parallel to the fibre axis [32]. This could suggest that further extension of the recombinant silk could have a similar result and thus have a significant effect on the final fibre mechanical properties.

Further interesting comparisons can be made between the structures of the bumblebee silk, the recombinant honeybee silk [30], and hornet silk gel films [5].

X-ray diffraction results obtained from undrawn hornet silk gel films were shown to have both coiled coil and β-sheet structural components with no specific orientation [5]. This is the same as found for the recombinant honeybee silk fibres [30]. Upon extension of the hornet silk gel films, little if any change was detected in the relative amounts of coiled coil and β-sheet protein segments, however, both have become highly orientated along the draw axis [5]. An orientation factor between 62% and 75% was found for the coiled coil segments while a much higher factor of 85% was found for the β-sheets. Closer examination of the X-ray data revealed that there were two different β-sheet regions present; one orientated and one random. The structure proposed for the bumblebee silk is consistent with that found for the drawn hornet silk gel films [5].

## 3. Materials and Methods

### 3.1. Silk

The silk was collected from *B. terrestris* (buff-tailed bumblebee) final instar larvae, obtained from hives provided by Koppert Biological Systems (Berkel en Rodenrijs, The Netherlands). Larvae that had just commenced spinning were held gently with a pair of tweezers and the spinnerette of the bumblebee larvae was gently bumped against a slowly spinning wheel. Extruded silk stuck to the reel and was able to be drawn from the insect. The silk fibres used in this study were reeled at 0.10 and 0.14 cm/s.

### 3.2. Microscopic Observations

Secondary electron images were obtained at low voltage using a Schottky Emission Variable Pressure Scanning Electron Microscope (SEM, Hitachi, Tokyo, Japan). The samples were mounted on an aluminium stub using conductive carbon tape. Imaged samples were coated with 4 nm of platinum—palladium using a Cressington 208 HRD high resolution sputter coater (Cressington Scientific Instruments, Watford, UK). Fibre diameter measurements were made using measureIT version 5.0 (Olympus Soft Imaging Solutions GmbH, Münster, Germany). EDX analysis was carried out on uncoated samples using a Vortex-EM Si drift detector (SII NanoTechnology, Northridge, CA, USA).

### 3.3. Raman Spectroscopy

Raman spectra were obtained using an inVia confocal microscope system (Renishaw, Gloucestershire, UK) with 514 nm excitation from an argon ion laser through a ×50 (0.75 na) objective. Incident laser power, as measured using an Nova power meter fitted with a PD300-3W head (Ophir, Jerusalem, Israel) was 4.5 mW and coaxial backscatter geometry was employed. The spectral resolution was ~1 cm^−1^. The Raman shifts were calibrated using the 520 cm^−1^ line of a silicon wafer. Survey spectra were collected over the range 3200 to 100 cm^−1^ and for fibres averaged over at least five scans, each with an accumulation time of 20 s.

Spectra of fibres were obtained from areas removed from cross-over points or areas where film was not present. Fibres were aligned parallel to the direction of laser polarization with the use of a rotating stage. For the polarization study the laser polarization was rotated using a ½ wave plate while the spectrometer was fitted with a polarization analyser consisting of a polarizer and a ½ wave plate. The microscope stage defines the *xz* plane and the fibre under analysis was aligned parallel to the *z* axis. Polarized spectra are identified as I_jk_ where j and k represent the laser and analyser orientations, respectively. Spectra were collected in static mode over the range 1840 to 1348 cm^−1^ for the four possible polarizer orientations.

Data manipulation was carried out using Grams AI 8.0 spectroscopy software. In all cases, the final spectra used for the analysis were averages of spectra collected from at least five different areas. No smoothing was carried out. Survey spectra were normalized on the C–H deformation vibration at 1450 cm^−1^ which is not sensitive to protein conformation. Spectral deconvolution was carried out by first identifying band components from the second derivative spectra obtained using the Savitzky-Golay method [43]. Fits were based on the usage of a minimal number of band components, each represented by variable mixture of Gaussian and Lorentzian functions [15]. All peak heights were limited to the range greater than or equal to zero. In the initial fitting steps, the band centres were only allowed to vary by ±5 cm^−1^ from the frequency determined by the second derivative spectra. In the final refinements all parameters were allowed to vary unconstrained. A linear baseline defined by the intensity at 1765 cm^−1^ was utilized with additional peaks being fit well past the spectral region of interest on the low wavenumber side. Orientation calculations were carried out using Matlab R2010a.

## 4. Conclusions

The Raman analysis of hand drawn *B. terrestris* silk enabled the identification of poly(alanine), poly(alanine-glycine), phenylalanine, tryptophan, and methionine, which is consistent with the results of previous amino acid analysis. From spectral deconvolution of the amide I band, the dominant protein conformation was found to be coiled coil (73%) while the β-sheet content of 10% is, as expected, lower than those reported for hornets and ants.

Polarized Raman spectra revealed that the coiled coil protein segments were highly aligned along the fibre axis. In this arrangement, the peptide C=O bonds are also roughly aligned in this way. The molecular chains of the β-sheet and random coil segments were also found to be aligned along the fibre axis. For the β-sheets this means that their peptide carbonyl groups are roughly perpendicular to the fibre axis. A structural model for the *B. terrestris* silk fibre is proposed based on these results.

The peptide C=O orientation distribution is compared to those of other natural and recombinant silks. Previous attempts to mimic bee silk fibres using recombinant materials, where structural units (coiled coils and β-sheets) were aligned using a post fibre fabrication process, did not mimic the orientation of the structures that we observed in the native bee silk.

## Figures and Tables

**Table 1:** Raman bands and their tentative assignments for the hand drawn *B. terrestris* silk fibre.

Frequency (cm^−1^)	Relative Intensity ^1^	Tentative Assignments ^1^
1673	w, sh	Amide I, β-sheet
1651	s	Amide I, coiled coil
1605	vw	ν(C=C) aromatic ring in F
1576	vvw	ν(C=C) aromatic ring in F and W
1554	vw	ν(C=C) aromatic ring in W
1457	m	δ(CH_2_) and δ(CH_3_) in poly(A) and poly(AG)
1333	w, sh	δ(CH)
1309	m	Amide III, coiled coil
1277	vw, sh	Amide III, random coil
1163	w	ν(C–C)
1123	w	ν(C–C) and ν(C–N)
1106	w	ν(C–C) and poly(A) (νC_α_–C_β_ and ρC_β_H_3_)
1084	vw	ν(C–C) skeletal random coil, β-sheet
1046	m	ν(C–C) skeletal
1006	vw	ν(C=C) aromatic ring breathing in F and W
981	vw	ρ(CH_3_)
908	m	ν(C–C) skeletal, poly(A), coiled coil
758	w	ρ(CH_3_) and skeletal bend in poly(A)
715	w	ν(C–S) M, *trans*
645	vw, br	ν(C–S) M
544	vw, sh	Skeletal bending
527	w	Skeletal bending in poly(A)
457	vw	Skeletal bending
415	vw	Skeletal bending
378	vw	Skeletal bending
229	w	Skeletal bending

^1^ ν, stretch; δ, bend; ρ, rock; s, strong; m, moderate; w, weak; v, very; sh, shoulder; br, broad; A, alanine; AG, alanine-glycine; M, methionine; F, phenylalanine; W, tryptophan.

**Table 2:** Amide I band deconvolution component parameters and assignments for the hand drawn *B. terrestris* fibre.

Band Position (cm^−1^)		Height (Counts)	Width (cm^−1^)	% Lorentzian	% Area	Assignment
2nd Derivative	Curve Fit					
1692	1691	722	21	99	4	β-turn
1673	1675	1766	28	32	10	β-sheet
1652	1651	12,326	23	100	73	coiled coil
1633	1633	2222	30	38	14	random coil

## Supplementary Materials

Supplementary materials can be found at http://www.mdpi.com/1422-0067/17/7/1170/s1.
