# Supplementary material for: Structural Analysis of Hand Drawn Bumblebee Bombus terrestris Silk
Source: Int J Mol Sci. 2016 Jul 20;17(7):1170. doi: 10.3390/ijms17071170 (PMC4964541; doi:10.3390/ijms17071170)
Supplement: Supplementary file 1 [file ijms-17-01170-s001.pdf]

Supplementary Materials: Structural Analysis of Hand Drawn Bumblebee *Bombus terrestris* Silk

Andrea L. Woodhead, Tara D. Sutherland and Jeffrey S. Church

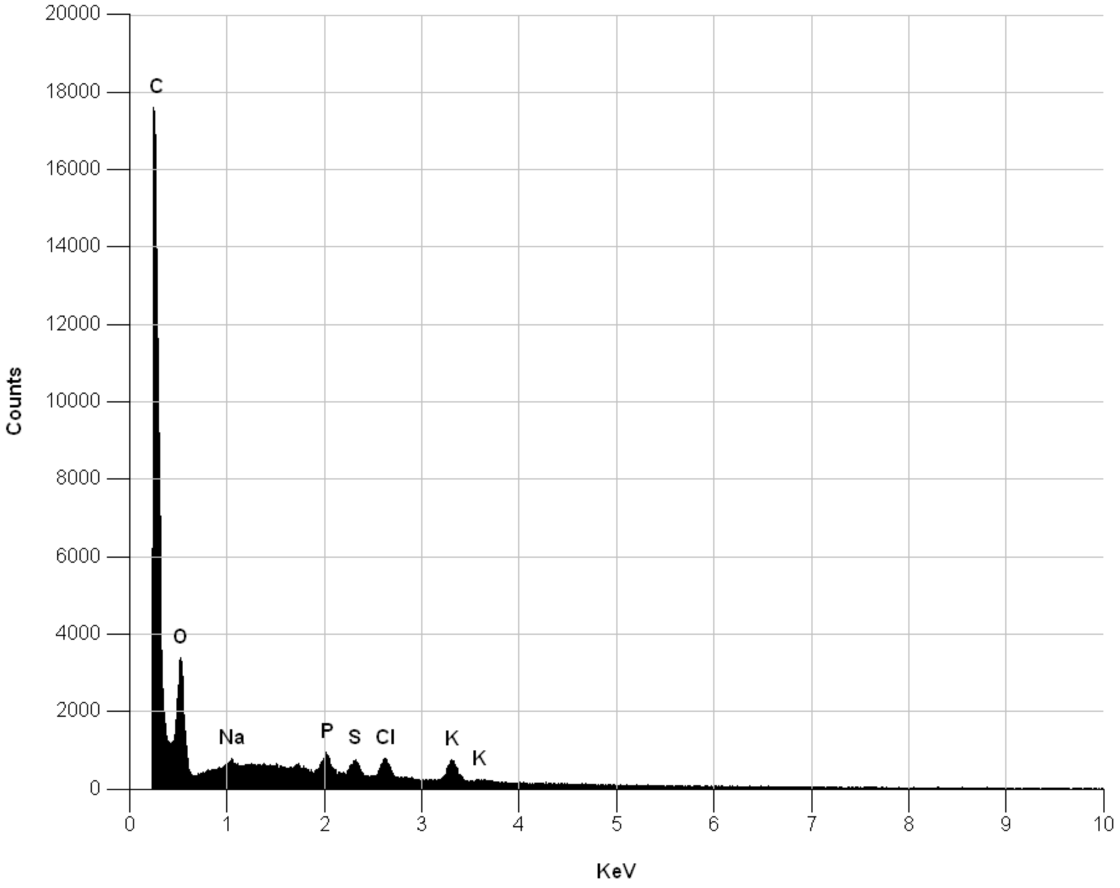

Figure S1. EDX spectrum obtained from the *B. terrestris* silk fibre hand drawn at 0.14 cm/s shown as Figure 1a,b.

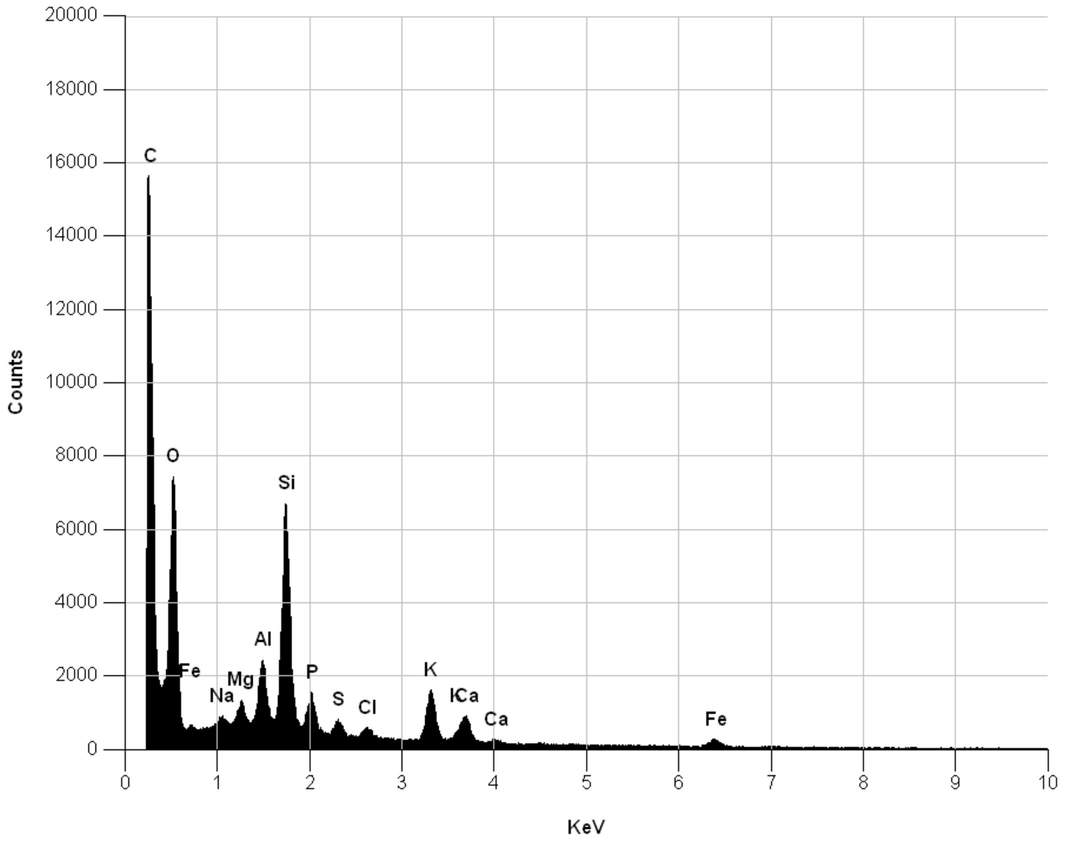

Figure S2. EDX spectrum obtained from the *B. terrestris* silk fibre hand drawn at 0.10 cm/s shown as Figure 1c.
